# The “reverse breast–oesophagus syndrome”: metastatic carcinosis of breast in oesophageal cancer

**DOI:** 10.1259/bjrcr.20150510

**Published:** 2016-11-02

**Authors:** Anjuna Reghunath, Yatish Agarwal, Avneet Singh Chawla, Mahesh Kumar Mittal, Swarna Gupta

**Affiliations:** ^1^Department of Radiodiagnosis, Vardhman Mahavir Medical College and Safdarjung Hospital, New Delhi, India; ^2^Department of Surgery, Vardhman Mahavir Medical College and Safdarjung Hospital, New Delhi, India

## Abstract

A “breast–oesophagus syndrome” has been described previously, wherein breast carcinoma metastasizes to the inner layers of the oesophagus. The entity is extremely rare, but rarer still is metastatic breast carcinosis from oesophageal cancer (EC), a clinical event that might be termed as “reverse breast–oesophagus syndrome”. Considered as the sixth most lethal malignancy, 50% EC patients present with metastatic disease. However, they rarely ever metastasize to the breast. For that reason, a malignant breast mass, which develops following EC, is often thought of as a second malignancy. We report a 62-year-old female who had EC, who was treated with oesophagectomy 2 years ago, and represented with a painful left breast mass. Radiological evaluation revealed suspicious findings (breast imaging-reporting and data system score of 4C), while cytology demonstrated squamous pearls, consistent with metastatic squamous cell EC, which probably disseminated to the breast at the time of surgery. She was treated with local excision of the breast mass, which is the treatment of choice in isolated metastasis to the breast. Such an unusual presentation reminds us that, in any “radiologically suspicious” breast lesion in patients with a history of carcinoma of the oesophagus, the possibility of breast metastasis must not be negated.

## Clinical presentation

**Figure 1.**
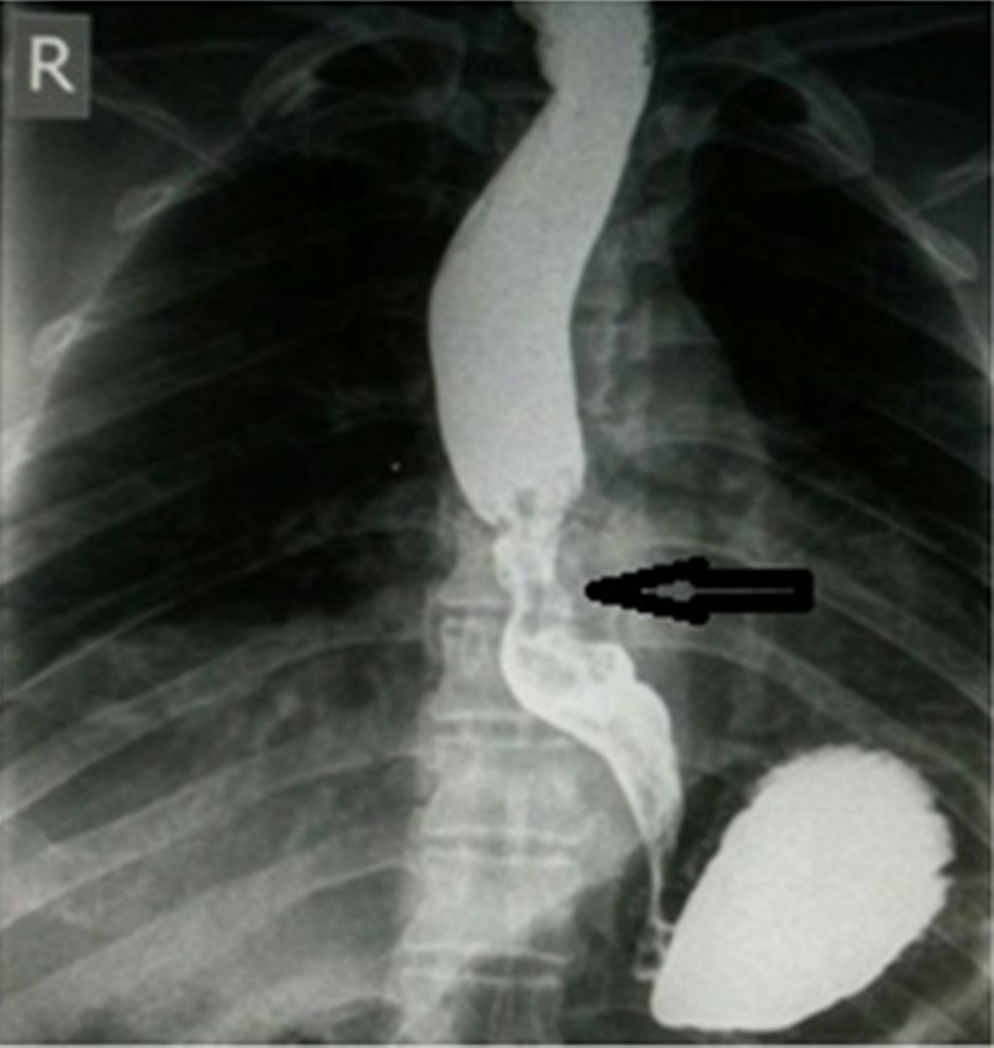
Barium swallow reveals the presence of a stenotic area with mucosal irregularity (arrow) in the mid-thoracic part of the oesophagus, distal to the carina.

A 62-year-old female patient presented to us in October 2015 with a painful left breast lump that had increased progressively in size over the past 8 months. She had undergone a surgical resection for mid-thoracic oesophageal carcinoma (EC) in 2013. At that time, she had complaints of dysphagia, retrosternal pain and weight loss, and her barium swallow had revealed a stricture with marked irregularity in the mid-thoracic part of the oesophagus, distal to the carina (Figure 1). Upper gastrointestinal endoscopy found a 6 cm mass, which was associated with mucosal irregularity and narrowing of the oesophageal lumen, 25 cm from the incisor teeth. Endoscopic punch biopsy confirmed the lesion to be squamous cell EC. The patient was taken up for oesophagectomy with a gastric pull-through operation, after being staged as T2N1M0 following staging work-up. The resected specimen was histologically found to be an invasive, moderately differentiated, squamous cell carcinoma punctuated by areas of poor differentiation. The resection was followed by four cycles of chemotherapy with 5-fluorouracil and cisplatin. Follow-up barium swallow was normal. Since her recovery, the patient did well until she developed a lump in her left breast. However, the patient refused to seek medical advice for a span of 8 months, as it was not painful until then. On examination, this was a firm 3 × 2 cm mass just beneath the nipple, which was tender and immobile. Axillary lymph nodes were not palpable bilaterally.

## Imaging findings

**Figure 2.**
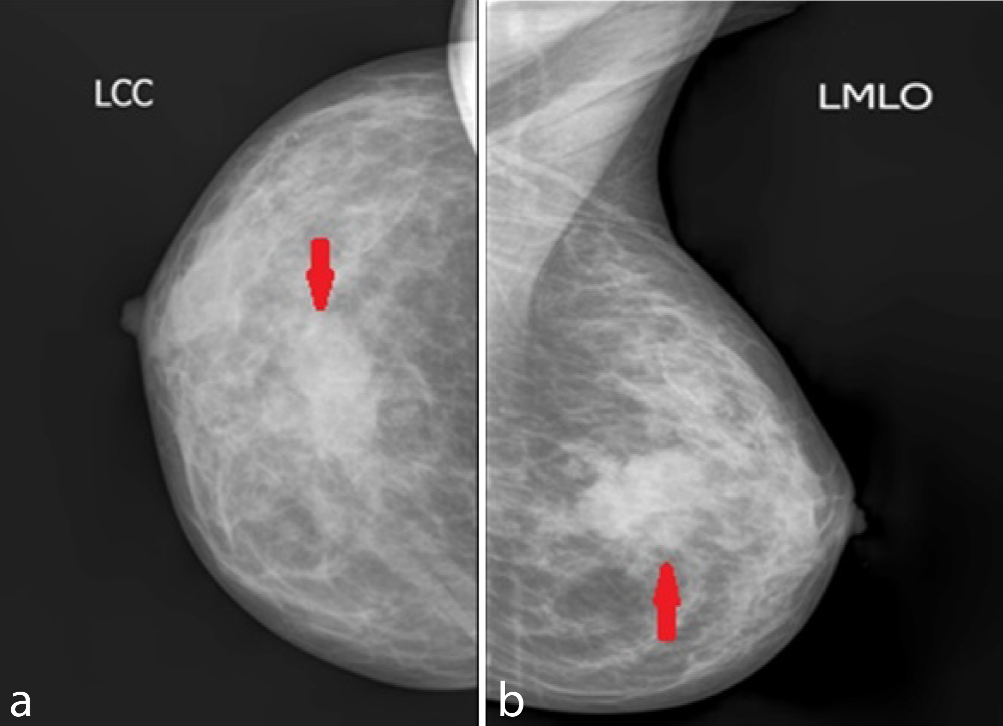
Mammography demonstrating a hyperdense lesion(arrows) with indistinct margins and surrounding architectural distortion on craniocaudal (a) and mediolateral oblique (b) views.

First, we put her through mammography, a basic screening test in late middle-aged females with a breast mass. Mammography demonstrated that the patient had a retroareolar, hyperdense, irregular mass lesion that had indistinct margins and was associated with surrounding architectural distortion (Figure 2a,b). No micro- or macrocalcification was discernible within the lesion.

**Figure 3.**
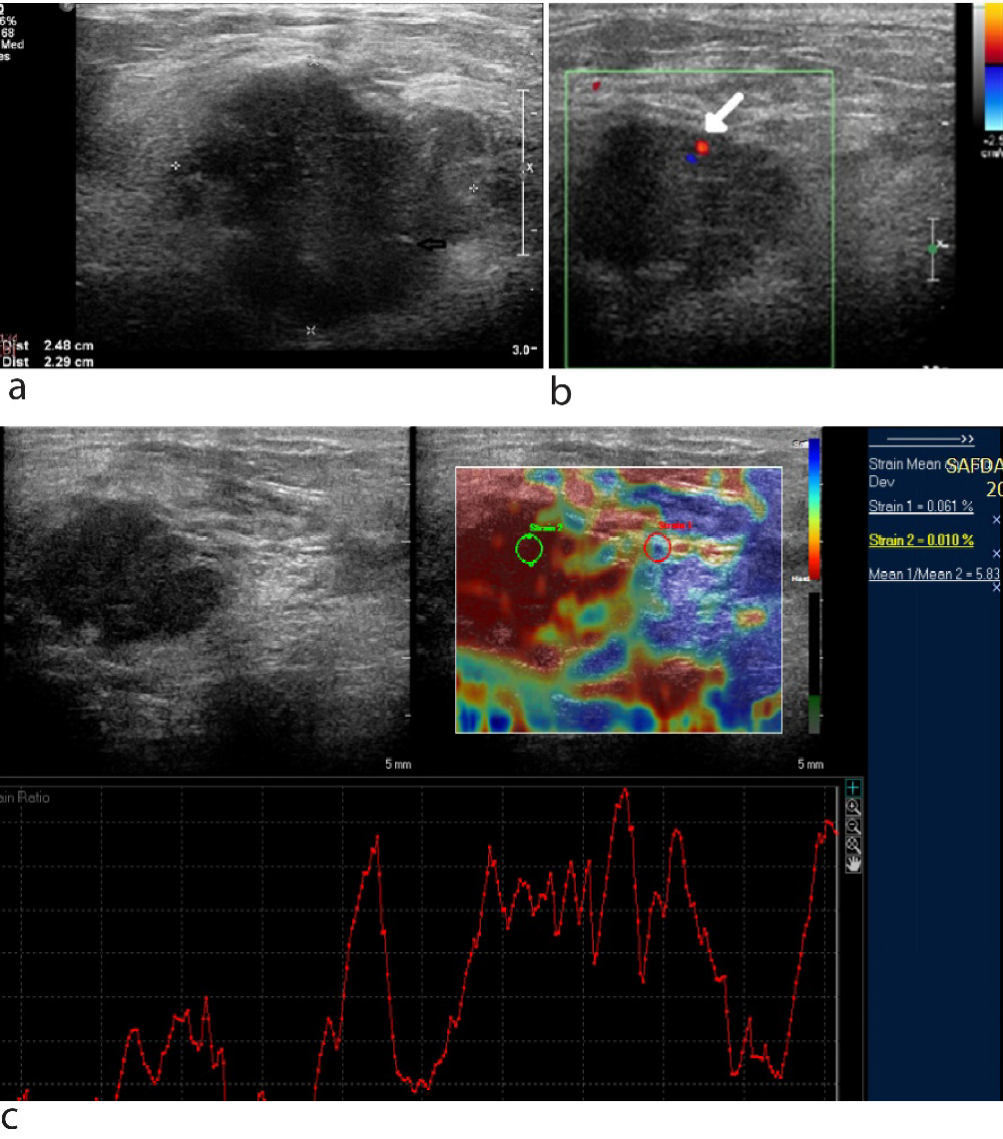
Ultrasound of the breast (a) showing a hypoechoic lesion with an irregular shape, angulated margins and macrocalcification (arrow), measuring 2.5 × 2.3 cm. The lesion is seen to abut the pectoralis major muscle but not infiltrate it. On colour Doppler examination of the breast lesion (b), internal vascularity (arrow) was noted. Assessment by strain elastography (c) revealed a stiff lesion, as indicated by the red colour on the colour-coded map of strain elastography. The regions of interest were selected in the preset shape of ellipse and the fat to lesion strain ratio with respect to the regions of interest was calculated to be 5.83.

On breast ultrasound, the lesion was hypoechoic; measured approximately 2.5 × 2.3 cm, had an irregular shape, angulated margins, demonstrated a speck of soft calcification without posterior acoustic shadowing and was abutting the pectoralis major muscle, but not infiltrating it (Figure 3a). The mass also did not reveal any posterior acoustic shadowing. The skin, nipple–areola complex and underlying muscle were not involved and thus we categorized the lesion as breast imaging-reporting and data system 4C (50–95% suspicion of malignancy), raising the possibility of a second primary malignancy in the breast and metastasis from the prior EC as the two differential diagnoses, the former being the more common scenario. On colour Doppler, the mass had internal vascularity (Figure 3b). Both axillae were normal. On strain elastography (SE), the mass had a Tsukuba elasticity score of 4 and strain ratio of 5.83, which was indicative of malignancy (Figure 3c).

**Figure 4.**
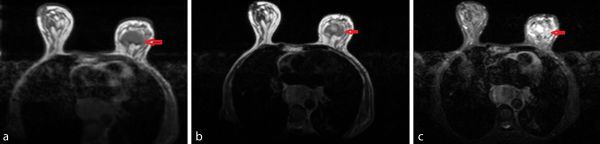
*T*_1_ weighted pre-contrast axial section of bilateral breasts (a) showing an irregular, hypointense mass lesion in the left breast in the retroareolar position (arrow). *T*_2_ weighted (b) and short tau inversion-recovery (c) images of axial section of the breasts demonstrating a heterogeneously hypointense lesion on the left side (arrows).

**Figure 5.**
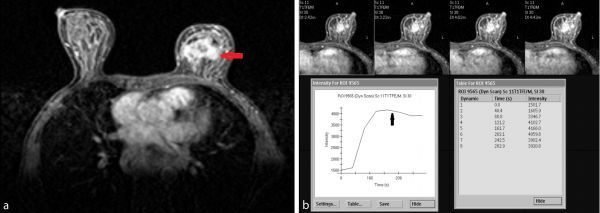
Axial post-contrast *T*_1_ weighted image (a) of bilateral breasts revealing the left-sided mass lesion with irregular margins and heterogeneous enhancement (arrow). The time-intensity kinetic curve obtained from the dynamic *T*_1_ weighted post-contrast scan of the breast lesion (b) revealing Type 2 plateau or indeterminate curve (arrow) showing progressive enhancement without any significant washout of contrast.

We performed an MRI of bilateral breasts for complete evaluation of the lesion to exclude any multifocal, multicentric disease, and for comprehensive evaluation of the axilla. *T*_1_ weighted pre-contrast scan showed an irregular, hypointense mass lesion in the left breast in the retroareolar position (Figure 4a). It was heterogeneously hypointense on *T*_2_ weighted and short tau inversion-recovery (STIR) images (Figure 4b,c). Dynamic contrast-enhanced MRI revealed an irregular lesion with heterogeneous enhancement on post-contrast *T*_1_ weighted imaging (Figure 5a) and Type 2 curve (plateau or indeterminate) on kinetic assessment (Figure 5b), all pointing to the possibility of a malignant lesion.

**Figure 6.**
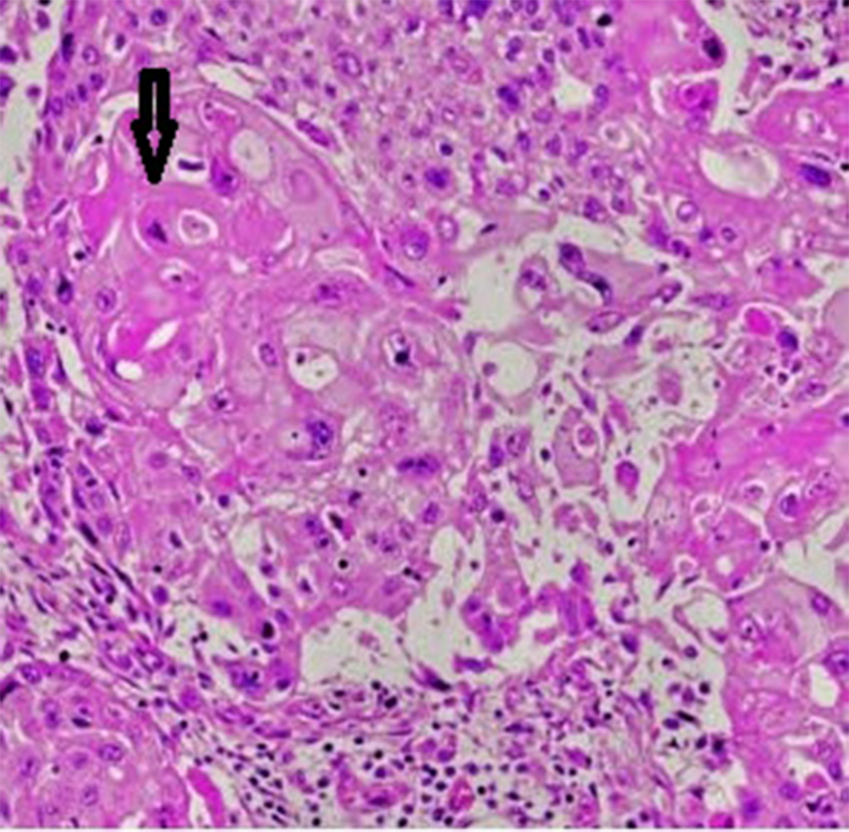
Histological sample of breast tissue after biopsy and haematoxylin and eosin staining showing the presence of squamous cells with keratin pearls (arrow) on 40x magnification.

A Trucut biopsy of the mass was carried out. Histopathologically, it demonstrated squamous cells with high nucleus/cytoplasm ratio, marked nuclear pleomorphism and keratin pearls, diagnostic of a squamous neoplasm (Figure 6). Immunohistochemistry was negative for oestrogen receptor, progesterone receptor and human epidermal growth factor receptor 2.

The patient was put through a detailed metastatic work-up, including a thoracic and abdominopelvic CT scan. She was found to have no other lesion and hence we diagnosed her as a rare case of isolated metastatic breast carcinosis from oesophageal squamous cell carcinoma with temporal heterogeneity.

## Differential diagnosis

### Primary carcinoma of breast

With a breast imaging-reporting and data system score of 4C, and considering the age of the patient, we kept in mind the differential diagnosis of a primary infiltrating ductal carcinoma of the breast, which has a more common incidence.

Even though the mass did not demonstrate some of the classic signs of malignancy, such as infiltration of the skin, subcutaneous tissue, nipple–areola complex or the underlying pectoralis major muscle, the mammographic presence of a hyperdense, irregular mass with indistinct margins and surrounding architectural distortion made primary breast malignancy a distinct possibility.

The ultrasound features of a hypoechoic, irregular lesion and internal vascularity on colour Doppler, favoured primary breast malignancy, although the signs of posterior acoustic shadowing and surrounding tissue infiltration were absent. The sonoelastography findings of Tsukuba score 4^1^ and strain ratio >3^2^ were inclined majorly in favour of the lesion being malignant.

On MRI, a classic primary breast cancer description is that of an irregular, infiltrating, hypointense lesion on *T*_1_ and *T*_2_ weighted, and STIR images, which enhances heterogeneously on administration of contrast. Kinetic curve assessment would depict Type 2 (plateau) or 3 (washout) curve and the lesion in question matched these classic features.

### Nodular sclerosing adenosis

This fibrosing variant of fibrocystic disease, which is common in the perimenopausal age group, was suspected, as it simulates carcinoma of the breast radiologically and even on cytology at times. On mammography, it presents as a hyperdense, irregular lesion with irregular microcalcification. On sonography, it appears as a hypoechoic, irregular mass with surrounding architectural distortion without internal vascularity on colour Doppler. SE may give a false-positive result with features of a stiff lesion (Tsukuba score 4) with strain index > 3 owing to fibrosis. However, MRI could reveal an irregular lesion with heterogeneous contrast enhancement and misleading dynamic parameters of rapid initial contrast enhancement and washout or plateau dynamic curves.^3^

### Tuberculous mass of breast

Considering the endemicity of tuberculosis in India and perimenopausal age of the patient, sclerosing variety of tuberculosis of the breast was the third differential diagnosis we had borne in mind. The mammographic findings of sclerosing tubercular mastitis consist of a homogeneous, irregular, dense mass, fibrous septa and retraction of the nipple. Ultrasound correlation often shows an increase in the echogenicity of the breast parenchyma without a definite mass lesion or internal vascularity. SE would show a soft lesion (Tsukuba score 1–3) with strain ratio < 3, indicating a benign lesion. However, the findings on MRI are very non-specific. Some reports state that MRI is only useful in evaluating the extramammary extent of the lesion.^4^

## Treatment and prognosis

The prognosis of metastatic EC is rather poor with a median survival of 6 months.^5^ A chemotherapeutic regimen may be initiated if the metastases exists in multiple organs. However, if the metastasis is solitary and lodged singularly in the breast, surgical excision of the lesion is the preferred treatment, even if beset with poor survival rates.^6^ Our patient underwent local excision of the breast lump, and follow-up visit after 1 month was uneventful.

## Discussion

Constituting about 7% of all gastrointestinal cancers, EC is the ninth most common malignancy and the sixth most frequent cause of cancer deaths globally.^7^ Histologically, it has two major subtypes: squamous cell type, which constitutes approximately 70% cases; and adenocarcinoma, which is the second, less common type.^8^ EC predominantly involves the upper and mid oesophagus.^6^ Distant metastasis occurs in 26% of locally advanced ECs during the initial 2 years of therapy and the most common sites of metastases are the lungs, liver and bone.^9^ However, metastasis to the breast is extremely rare, and only six such reports exist in the literature.^9^ The reverse phenomenon of metastasis of breast carcinoma to the mucosal and submucosal layers of the oesophagus is, however, far more common and has been described as the “breast–oesophagus syndrome”.^10^ The most common site of primary malignancy for metastatic carcinosis of the breast is the contralateral breast. The chances of extramammary malignancies metastasizing to the breast are rather low. Lymphomas, melanomas and rhabdomyosarcomas are the more likely villains.^9^ The incidence of extramammary malignancies metastasizing to the breast is estimated to be 0.5–5.1%.^5^ This low incidence of metastasis to the breast possibly relates to the presence of large amounts of fibrous tissue and relatively poor vascular supply in the breast, making the breast an unusual site for metastatic lesions.^11^ Females of younger age, who have greater vascular supply to the breast, are more liable to suffer metastatic lesions.^11^

The development of metastatic breast carcinosis following surgical resection of an apparently localized primary EC would, in all likelihood, be a fallout of a micrometastatic tumour spread at the time of surgery.^12^ The usual clinical presentation of a metastatic breast lesion is that of a single, painless, mobile, well-circumscribed mass sited in the upper quadrants of the breast.^9^

Making a radiological diagnosis of metastasis to the breast is no mean task, since the lesions have been described to present with varied appearances, ranging from benign to typically malignant masses.^9^ For example, on mammography, they may manifest as single or multiple masses, or even diffuse skin thickening, though the usual presentation is that of a solitary, irregular/well-defined, hyperdense lesion without spiculature in the upper outer quadrant.^6^ In this patient, the morphological appearance matched the latter, though the position was atypical. Ultrasound presentation of a metastatic lesion is usually that of a hypoechoic, well-circumscribed or irregular lesion with internal vascularity and without infiltration or posterior acoustic shadowing. Sonoelastography should demonstrate a stiff lesion (Tsukuba score 4 or 5) with strain ratio > 3, as in the case of any malignant lesion. In the presented case, the lesion was hypoechoic, possessed irregular margins without florid infiltration, had internal vascularity as well as Tsukuba score 4 with strain ratio of 5.83.

The suspicion was further intensified when *T*_1_ and *T*_2_ weighted, and STIR images revealed a hypointense, irregular lesion and dynamic contrast-enhanced MRI gave the picture of an irregular, heterogeneously enhancing lesion with Type 2 curve on kinetic assessment, which may be the presentation of any breast carcinoma, including metastasis. Thus, the definitive diagnosis rests squarely on histopathology.

This unusual clinical presentation of a progressively growing breast lump, which is firm and immobile on palpation, possessing imaging characteristics suspicious of malignancy and revealing a squamous cell neoplasm as histodiagnosis, in a previously detected/treated case of squamous cell carcinoma of the oesophagus, may be termed the “reverse breast–oesophagus syndrome”.

## Learning points

1. Possibility of metastasis to the breast should always be kept in mind, even in surgically treated EC patients.
2. High index of clinical suspicion is of utmost importance while dealing with such lesions, as they can be misdiagnosed as a benign mass or primary breast malignancy owing to their wide spectrum of radiological presentation.
3. The definitive diagnosis rests on histopathology, which if it reveals a squamous cell neoplasm, may be termed the “reverse breast–oesophagus syndrome”.

## Consent

Written informed consent was obtained from the patient for publication of this case report and the accompanying images. The anonymity of the patient has been maintained.
